# Heart Rate Variability During a Standard Dive: A Role for Inspired Oxygen Pressure?

**DOI:** 10.3389/fphys.2021.635132

**Published:** 2021-07-26

**Authors:** Pierre Lafère, Kate Lambrechts, Peter Germonpré, Ambre Balestra, Faye Lisa Germonpré, Alessandro Marroni, Danilo Cialoni, Gerardo Bosco, Costantino Balestra

**Affiliations:** ^1^Environmental, Occupational & Ageing Physiology Laboratory, Haute Ecole Bruxelles-Brabant, Brussels, Belgium; ^2^DAN Europe Research Division, Roseto degli Abruzzi, Italy; ^3^Laboratoire ORPHY, EA4324, Université de Bretagne Occidentale, Brest, France; ^4^Centre for Hyperbaric Oxygen Therapy, Military Hospital “Queen Astrid”, Brussels, Belgium; ^5^Environmental Physiology and Medicine Laboratory, Department of Biomedical Sciences, University of Padova, Padova, Italy; ^6^Physical Activity Teaching Unit, Motor Sciences Department, Université Libre de Bruxelles (ULB), Brussels, Belgium

**Keywords:** fractal, environmental stress, autonomic nervous system, nonlinear analysis, heart rate variability, self-contained underwater breathing apparatus, diving

## Abstract

**Introduction:** Heart rate variability (HRV) during underwater diving has been infrequently investigated because of environment limitations and technical challenges. This study aims to analyze HRV changes while diving at variable hyperoxia when using open circuit (OC) air diving apparatus or at constant hyperoxia using a closed-circuit rebreather (CCR). We used HRV analysis in time and frequency domain adding nonlinear analysis which is more adapted to short-time analysis and less dependent on respiratory rate (Sinus respiratory arrhythmia).

**Materials and Methods:** 18 males, 12 using OC (30 mfw for 20 min) and 6 using CCR (30 mfw for 40 min.). HRV was recorded using a polar recorder. Four samples of R-R intervals representing the dive were saved for HRV analysis. Standard deviation of normal-to-normal intervals (SDNN), square root of the mean squared differences between successive RR intervals (rMSSD), and average RR intervals (RR) in time-domain; low frequency (LF) and high frequency (HF) in frequency domain were investigated. Nonlinear analysis included fractal dimension (FrD).

**Results:** SDNN and rMSSD were significantly increased during descent and at depth with OC, not with CCR. Mean RR interval was longer at depth with OC, but only during ascent and after the dive with CCR. HF power was higher than baseline during the descent both with OC and CCR and remained elevated at depth for OC. The LF/HF ratio was significantly lower than baseline for descent and at depth with both OC and CCR. After 30 min of recovery, the LF/HF ratio was higher than baseline with both OC and CCR. Nonlinear analysis detected differences at depth for OC and CCR.

**Discussion:** Increased parasympathetic tone was present during diving. RR duration, SDNN; rMSSD, HF spectral power all increased during the dive above pre-dive levels. Conversely, HF power decreased (and the LF/HF increased) 30 min after the dive. Using FrD, a difference was detected between OC and CCR, which may be related to differences in partial pressure of oxygen breathed during the dive.

## Introduction

During self-contained underwater breathing apparatus (scuba) diving, a decreased heart rate seems a well-established steady-state response to pressure (Daniels and Grossman, 2003). Several influences likely combine during diving to change heart rate, and their individual effects are difficult to study. One mechanism of heart rate lowering is a change of activity of the autonomic nervous system (ANS). According to several authors, this change may be of primary importance and could even account for some diving accidents (Schipke and Pelzer, 2001; Mitchell et al., 2005; Pelliccia et al., 2005). It seems thus important to further assess diving related changes in heart rate and in their ANS triggers to enforce divers’ safety (Lundell et al., 2019; Schirato et al., 2020).

The cardiovascular system is challenged by various combinations of the direct effects of water immersion, thermal strain, exercise, depth pressure, hyperoxia, or apnea. Cold and hyperbaric pressure contributing to increase parasympathetic activity when diving in very cold water under constant hyperbaric pressure (Lundell et al., 2019). During thermo-neutral immersion hydrostatic pressure restrains the vascular compliance, which leads to a redistribution of blood volume from peripheral to central circulation (Norsk et al., 1985; Gabrielsen et al., 1993). During this condition of replete vasculature, the need for sympathetic activity maintaining smooth muscle tone in the vascular wall is lessened (Miwa et al., 1997; Mourot et al., 2008). On the other hand, the redistribution of regional blood flow is responsible for an increase in heart volume, stroke volume, and cardiac output (Norsk, 1992; Miwa et al., 1996; Shiraishi et al., 2002) accompanied by bradycardia, resulting from vagal activation. In addition to hydrostatic pressure *per se* and immersion, real diving (submersion) may exert its effects on the diver’s cardiovascular system by mechanisms, such as increased partial pressures of inhaled gases. Cardiovascular sympathetic influence is lowered by normobaric and hyperbaric hyperoxia (Lund et al., 1999; Gole et al., 2011) while conversely cold-water immersion causes an increased systemic sympathetic activity (Mourot et al., 2008).

The use of enriched air can result in oxidative stress affecting cardiorespiratory and vascular systems as it has been reported during CCR diving (Bosco et al., 2018). In general, in chronic disorders associated with oxidative stress (diabetes, cardiovascular (ischemic) disorders, etc.), a depression of aerobic metabolism is observed, reflected by low HRV (Valko et al., 2007; Sarsour et al., 2009; Cipak Gasparovic et al., 2017). On the contrary, high intensity of redox reactions (more efficient autonomic regulation) is usually reflected by high HRV (Yelisyeyeva et al., 2012).

However, regarding diving conditions, the possible effects of acute exposure to oxygen-enriched diving oxidative state in humans remain unclear and only few studies addressed the effects of diving on ANS (Chouchou et al., 2009; Zenske et al., 2020a). Recently, Zenske et al. showed that parasympathetic activation was increased during nitrox diving, and this increase was marked enough to reduce heart rate during the dive (Zenske et al., 2020b).

As far as we know, the bulk of knowledge up to now comes from isolated sinus node pacemaker preparation, animal studies (frog, eel, mice, etc.), or studies in healthy humans immersed up to the neck, “head out immersion.” Data concerning ANS responses to real scuba diving are only available from one study in a swimming pool (Schipke and Pelzer, 2001) and three in open seawater (Chouchou et al., 2009; Lundell et al., 2019; Zenske et al., 2020a). All have pointed that during the dive, heart rate variability (HRV) displayed an increased high frequency (HF) power spectral density, which is believed to reflect parasympathetic influence on heart rate modulation. Conversely, HF power was lowered and power in low frequencies (LF) of variability appeared higher during the recovery period. This latter HRV profile is considered as reflecting larger vascular influences, and hence at least in part peripheral sympathetic tone.

During a standard air dive, Dalton’s Law of Physics implies that the partial pressure of oxygen (PpO_2_) increases during the descent phase, remains elevated during the bottom phase, and decreases again with decreasing environmental pressure during the ascent phase at the end of the dive. In contrast, during (electronic) closed-circuit rebreather (CCR) diving, a certain PpO_2_ is selected by the diver and remains constant through automatic addition of oxygen by the diving apparatus.

CCR diving is becoming more and more popular. We therefore investigated “standard” dive profiles while minimizing as many of the aforementioned interfering factors as possible. First, we wanted to confirm that HRV descriptors are able to assess ANS-mediated changes in heart rate modulation. We also wanted to evaluate the effect (if any) of sustained hyperoxia (with CCR) versus variable hyperoxia (with OC) on changes in HRV parameters.

## Materials and Methods

### Subjects

After Ethics Committee approval (B200-2020-088) and individual written informed consent, 18 male experienced divers volunteered for the study. They were at least certified as “autonomous diver” according to European norm EN 14153–2 or ISO 24801-2, and each of them counted at least 50 logged dives. They were selected from a large sports/technical diver population to obtain a group of homogenous age (30–40 years), similar body composition (BMI between 20 and 25), and similar health status: non-smokers with regular but not very high physical activity (aerobic exercise one to three times a week).

### The Dive

All the subjects were assessed as fit to diving before entering the study. None had a history of the previous cardiac abnormalities and none of them were on any cardiovascular medication. According to their diving qualification, subjects were scheduled to perform either an open-circuit dive (group 1 = 12) or an electronic closed-circuit rebreather (eCCR) dive (group 2 = 6) under direct underwater supervision of at least one of the authors in every group to avoid uncontrolled movements, position changes (divers were asked to keep a flat streamline position), and other possible unwanted actions underwater. They were instructed not to dive within 72 h before the experimental dive and not to drink any alcoholic or caffeinated beverages for 4 h before the dive.

### Open-Circuit Dive

Each diver of group 1 performed a 30 m depth dive into fresh water (mfw) for 20 min in a pool environment (Nemo33, Brussels, Belgium) in a 33°C water, which normally do not require any thermal protection suit. However, each diver wore a 5 mm neoprene suit. This depth-time profile is embedded within the “no-decompression limits” of the US Navy dive tables, 2008 edition (NAVSEA, 2008). Descent speed was 15 meters per minute and ascent speed to the surface 10 meters per minute, with no safety stop (none required according to the NAVSEA, 2008 dive table used).

### Closed-Circuit Dive

Because CCR diving was not allowed in the Nemo 33 pool CCR divers performed a 30 m depth dive in a flooded quarry with fresh water. Using a Megalodon rebreather (InnerSpace System Corp, Centralia, Washington, United States), a constant partial pressure of oxygen was set at 1.3 ATA with air as diluent gas; this yielded an equivalent air depth (EAD) of 24.2 mfw. Bottom time was set to 40 min so that the depth-time profile also lies within the same “no-decompression limits” (NAVSEA, 2008). Nonetheless, for safety reasons, a 6 min safety stop at 6 m was added. Descent and ascent speeds were kept similar to those in the pool experiment. As the water temperature was measured at 17°C (Scubapro-Uwatec digital depth gauge, Hallwil, Switzerland), divers wore a trilaminate drysuit equipped with dry gloves, a 5 mm neoprene semi-dry hood with face and neck seals so that only the mouth area was exposed to the water. They also wore a Thinsulate thermal underwear and a battery-powered heated vest system (Silent Planet, Portland, Dorset, England).

Before and after the CCR dive, cervical-supraclavicular area skin temperature was evaluated using a forward-looking infrared (FLIR) camera, confirming the absence of significant skin cooling during the dive (Pre-dive: 36.9 ± 0.5°C vs. post-dive: 37.5 ± 0.7°C; *p* > 0.05).

### Data Recording and Analysis

All data were obtained using a polar watch with chest belt-mounted sensor (Polar RS800 sd, Polar Electro, Oulu, Finland), a validated noninvasive device to measure heart rate (HR) and HRV (Radespiel-Troger et al., 2003). The polar belts were set while the volunteers were putting their gear on. The polar watches were either worn at the wrist (group 1) or placed in the breast pocket of the undergarment inside the suit (group 2). The quality of recording is not altered if the watch is in contact with water and does not depend on its positioning on the wrist of inside the breast pocket.

The sampling rate used was 1,000 Hz and the recording began immediately after the participants got dressed (i.e., 40 ± 5 min before the dive) and finished 30 min after the dive. During the period before and after the dive, the subjects were instructed to rest and sit quietly on benches along the water entry, and to not either talk or stand up.

**Figure 1:**
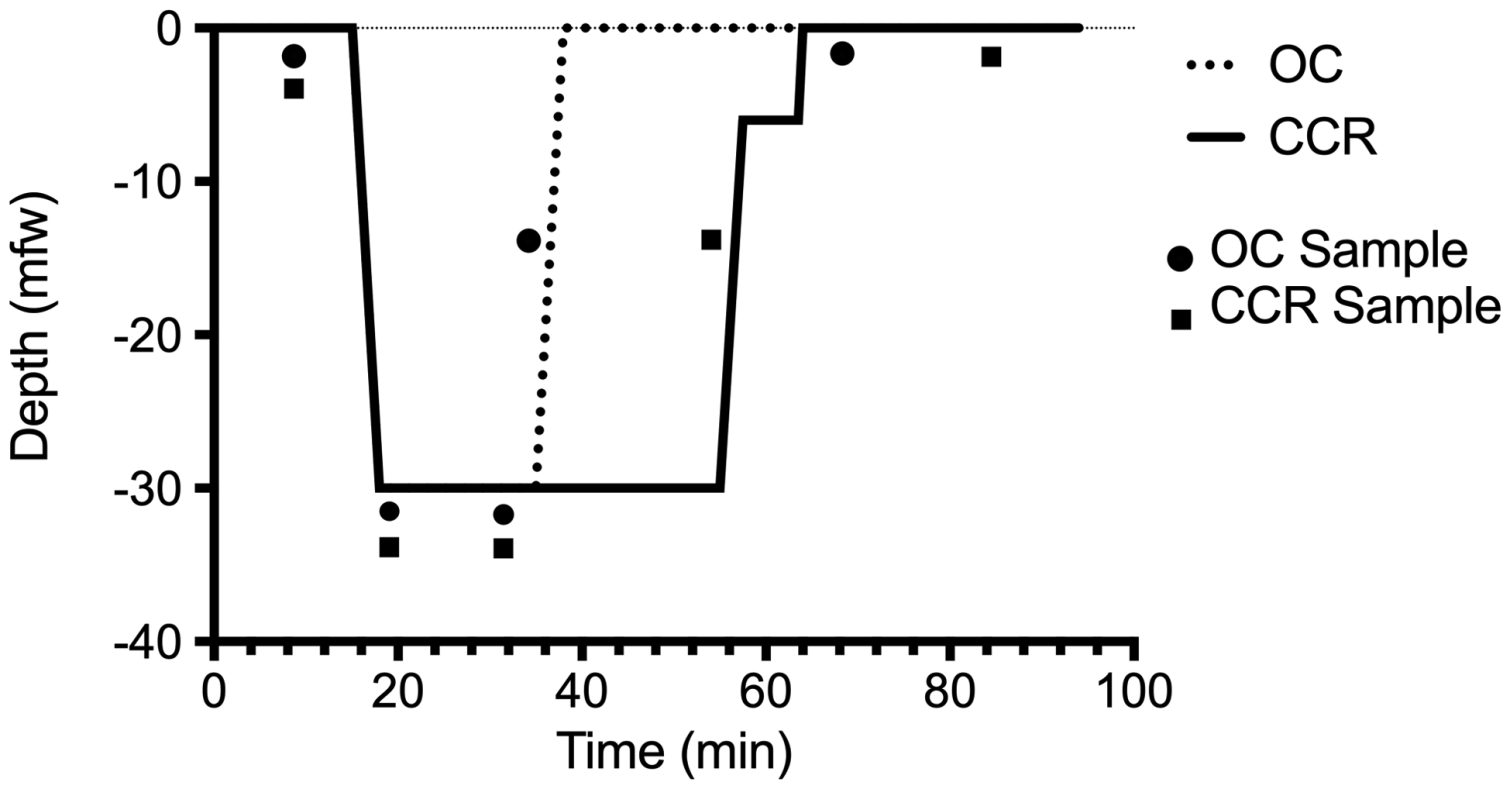
Dive profile. Open-circuit (OC) depth of 30 mfw for 20 min in a pool environment (Nemo33, Brussels, Belgium). Closed-circuit rebreather (CCR) depth of 30 mfw for 40 min. The dive was made with a constant oxygen partial pressure of 1.3 bar on an air diluent providing an EAD of 24.2 mfw. The two depth-time profiles are embedded within accepted “no-decompression limits” (NAVSEA, 2008).

Samples of 256 cycles were selected within each segment of the dive (Figure 1). The first sample was taken after a 10 min rest (baseline), the second just after submersion (descent), the third after 15 min at targeted depth (depth), the fourth was chosen during the ascent (ascent), and the fifth 30 min after emersion (recovery). R-R intervals were extracted and saved for subsequent HRV analysis. All the R-R intervals were edited initially by visual inspection to exclude all the undesirable beats (i.e., to ensure that each analysis for the segment was free of movement artifact and/or sharp transient in the signal due to premature beat), which accounted for less than 1% occurrence in every subject.

Global activity of the ANS and isolated sympathetic and parasympathetic branches activity were analyzed using the Kubios software HRV 2.0 (UKU, Kuopio, Finland) according three methods: time-domain, frequency domain, and nonlinear analysis. Time-domain measures were mean heart rate (HR; bpm), standard deviation of NN intervals (SDNN), and root mean square of successive RR interval differences (RMSSD). Frequency domain measures were absolute total power (*P*_tot_), the power of spectral components in the low (LF) and high frequencies (HF) along with the LF/HF ratio. Nonlinear analysis with Poincare plot was used to calculate short-term (SD1) and long-term (SD2) HR variability. In addition, the approximate entropy (ApEn), a measure of the complexity or irregularity of the signal, and the fractal dimension (FrD) were also calculated, using the ORTO science software 4.9.85 (Alive System Llc, Kemerovo, Russia) along with the short-term fractal-scaling exponent (*α*1) measured by the detrended fluctuation analysis (DFA) method (Henriques et al., 2020).

During scuba diving, a decrease in breathing frequency (Schipke and Pelzer, 2001), may shift the respiratory peak from the usual high-frequency band (HF) to the usual low-frequency band (LF; Brown et al., 1993). Therefore, to avoid the influence of respiratory sinus arrhythmia (Cavalade et al., 2015), we adapted the limits of frequency analysis: total spectral power (*P*_tot_: 0–0.4 Hz), high frequency (HF: 0.1–0.4 Hz), and low frequency (LF: 0–0.1). Power spectral density of HRV (Niskanen et al., 2004) was calculated according to these limits.

### Statistical Analysis

Standard statistical analysis was performed, including mean, standard deviation, and ANOVA for repeated measures to test the inter- and intra-subject variability after Kolmogorov-Smirnov test for normality using GraphPad Prism version 5.00 for Windows (GraphPad Software, San Diego California, Unites States). A threshold of *p <* 0.05 was considered statistically significant.

## Results

All set of data passed the Kolmogorov-Smirnov test allowing us to assume a Gaussian distribution.

The two groups of subjects were not different for age, BMI, and pre-dive “resting” heart rate (Table 1). Divers in the CCR group had a significant greater number of logged dives than the OC group (*p* = 0.0003). This is explained by the fact that the CCR group was exclusively comprised of dive instructors.

**Table 1:** Anthropometric data and time of RR interval sampling in the two diving groups.

	OC	CCR	*p* value
Age (years)	35 ± 3	35 ± 3	0.529
BMI (kg/m^2^)	21.7 ± 1.1	21.8 ± 1.2	0.751
Resting heart rate (beat/min)	102 ± 13	105 ± 34	0.787
Logged dive	202 ± 129	1,609 ± 1,083	0.0003
Baseline (minutes)	10.2 ± 0.5	10.2 ± 0.4	0.714
Descent (minutes)	17.3 ± 0.6	17.4 ± 0.6	0.735
Depth (minutes)	32.2 ± 0.6	32.3 ± 0.6	0.659
Ascent (minutes)	35.2 ± 1.1	54.2 ± 0.7	<0.0001
Recovery (minutes)	65.2 ± 1.1	83.3 ± 0.7	<0.0001

*OC, open-circuit scuba diving; CCR, closed-circuit rebreather. Times to ECG sampling periods are given according to a timeline where T0 corresponds to a fully equipped diver resting on a bench before the dive*.

The first three samples of R-R intervals (baseline, descent, and depth) were taken at similar times in the groups (*t*-tests: *p* > 0.1). The last two RR interval samples were taken later in the CCR group according to the design of the study (Figure 1). This was chosen to harvest the ascent sample (4) at a similar amount of inhaled nitrogen (bottom time of 20 min in OC versus 40 min in CCR). The recovery sample (5) was taken after a post-dive (out of water) period of 30 min.

**Figure 2:**
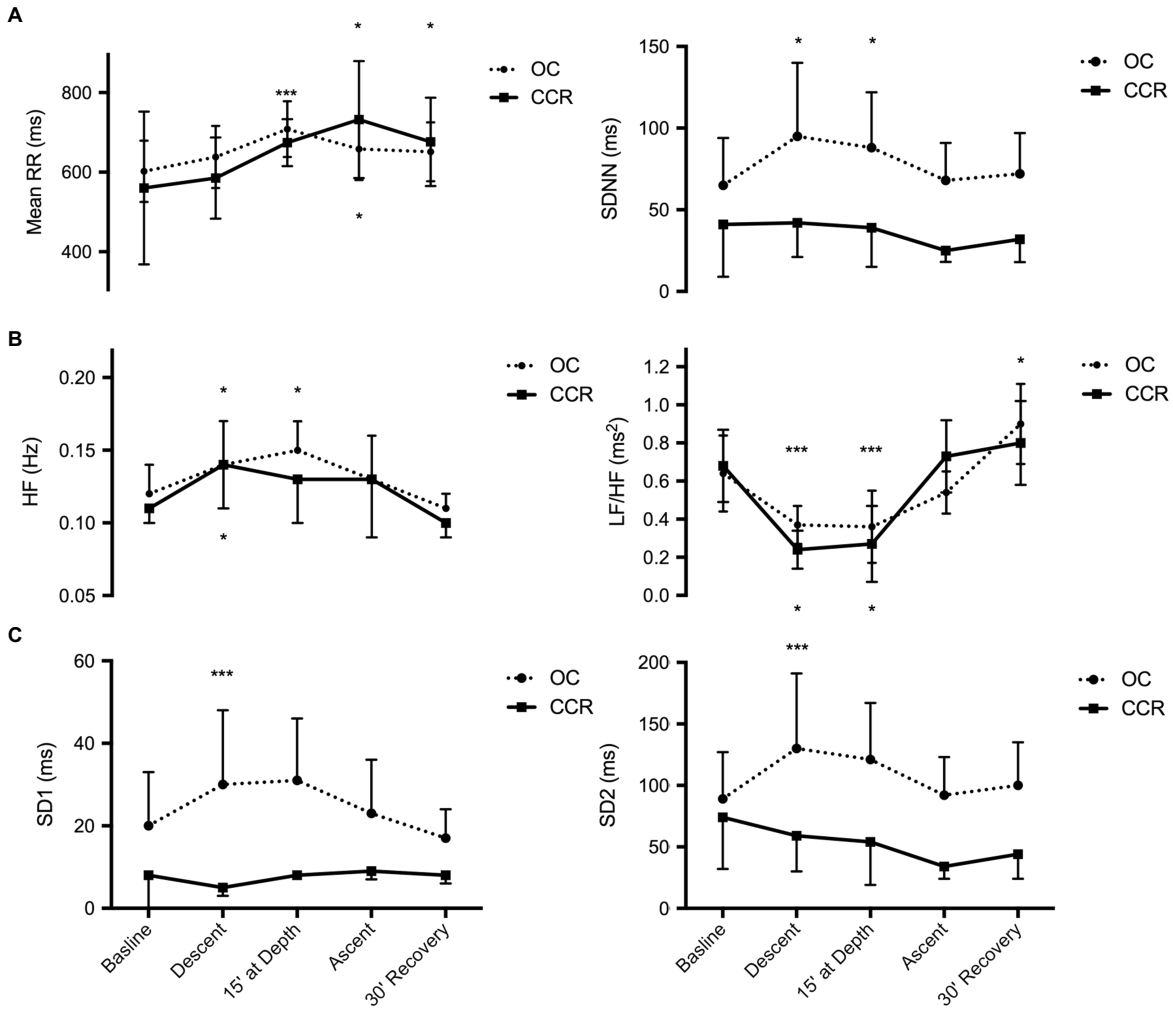
Analysis of heart rate variability. **(A)** Time-domain analysis; **(B)** Frequency domain analysis; and **(C)** Poincare plot. Mean RR, mean RR intervals; SDNN, standard deviation of normal-to-normal intervals; spectral power of HR variability; HF, high frequency and LF/HF, low frequency to high frequency ratio; SD1, beat-to-beat HR variability; and SD2, global HR variability. ^*^p < 0.05; and ^***^p < 0.001.

SDNN and rMSSD were higher than baseline during descent and at depth with OC (*p* < 0.05) but did not change with CCR as shown in Figure 2. On average, RR interval was higher than baseline at depth with OC and during the ascent with CCR.

The spectral HF power was higher than baseline during the descent with OC and CCR, and at depth during OC (*p* < 0.05). During post-dive recovery, HF spectral power had returned to baseline levels. The LF/HF ratio was lower than baseline during the descent and at depth with both OC and CCR (*p* < 0.01). After 30 min of recovery, the LF/HF ratio had returned to a higher than baseline value with both OC and CCR (*p* < 0.05).

During recovery, LF was higher than period and the dive (59.4 ± 15.6 vs. 45.4 ± 13.6) while HF was lower (62.1 ± 15.2 vs. 69.5 ± 11.1, *p* < 0.05).

With OC, SD1 and SD2 increased above baseline (*p* < 0.01) during the descent and were thereafter no more different from baseline. With CCR, SD1 and SD2 did not change significantly at any period.

**Figure 3:**
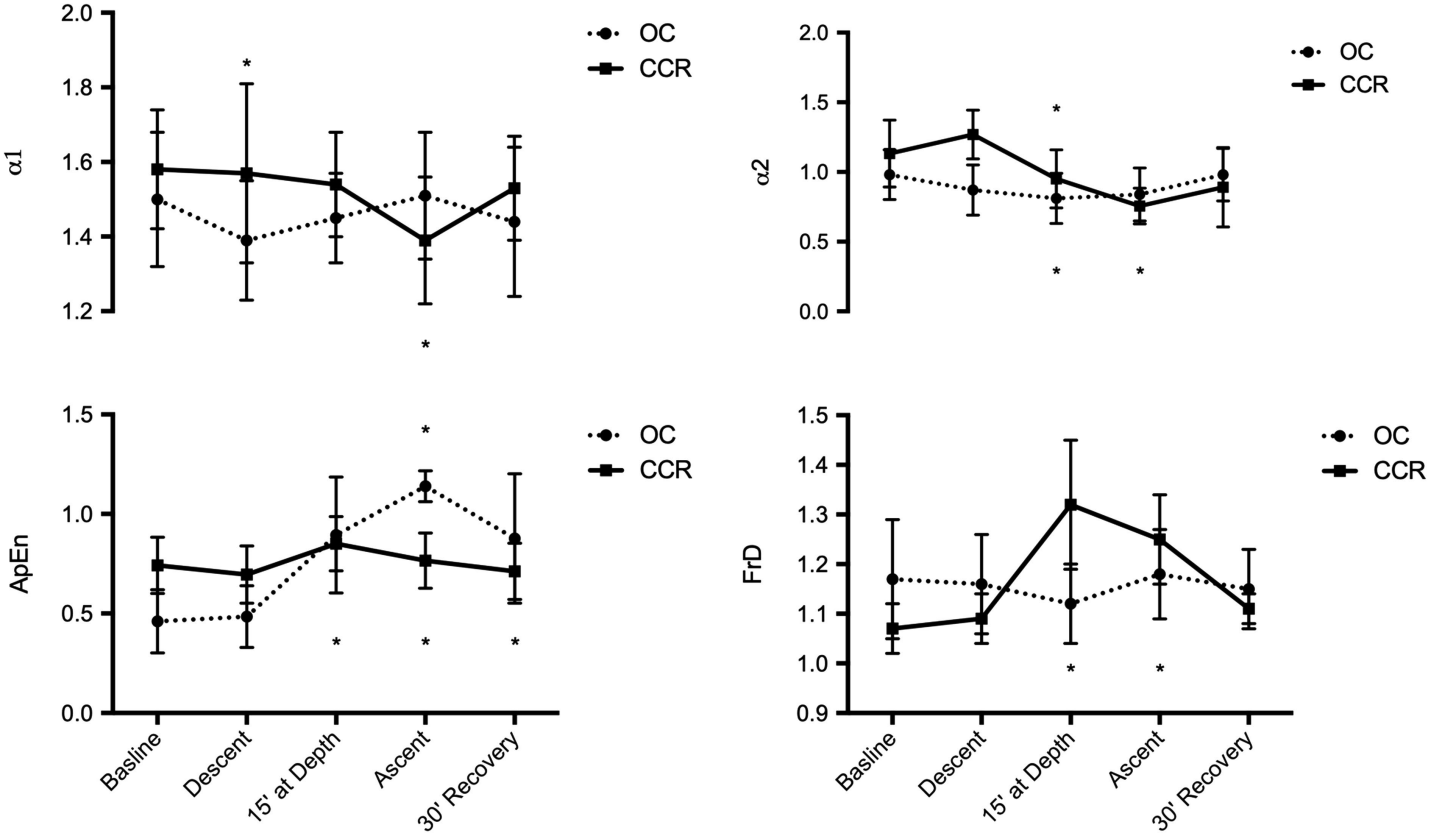
Fractal analysis of HR variability. α1 & α2, fractal-like scaling exponent; ApEn, approximate entropy; and FrD, fractal dimension ^*^p < 0.05.

FrD did not change with OC but increased significantly with CCR during the period at depth and during the ascent. This increase was significantly different from the OC group (*p* = 0.0002 and *p* = 0.008, respectively). In the CCR group, ApEn was also higher at depth, during the ascent and after the dive, than at baseline (*p* < 0.05). However, the difference with the OC group was not significant (*p >* 0.05; Figure 3).

## Discussion

Data of HRV during scuba diving in open water are presently only available from a limited number of studies (Chouchou et al., 2009; Lundell et al., 2019, 2021; Zenske et al., 2020a). Similar to our experience, most of these studies involved a small group of divers, with variable hyperbaric exposure and were not convincingly able to separately evaluate the roles of depth, thermal status, or oxygen exposure, which were shown to be important (Lundell et al., 2021). Considering the setting, we share most of these limitations.

The results of the first part of our study (OC dive) confirm HRV changes similar to those previously reported (Chouchou et al., 2009). These changes reflect an increase of parasympathetic influence on heart activity during the descent and the bottom phase, followed by a decrease of vagal activity in the post-dive period. Indeed, during the dive, the RR duration, the SDNN and rMSSD, and the HF spectral power all increased above baseline (pre-dive) levels. Conversely, the HF spectral power was lowered (and the LF/HF ratio increased) 30 min after the end of the dive. Such changes in autonomic modulation of heart activity may be elicited by several triggers, among which body position. Baseline and post-dive RR recordings were performed in sitting subjects wearing their diving suit. Upon descent and at depth, the subjects were mostly in prone position, which favors the arrival of blood from the lower limb and splanchnic vascular beds into the thorax and pulmonary vessels. This filling of the central vascular bed leads in turn to a higher cardiac vagal tone (Bahjaoui-Bouhaddi et al., 2000; Mourot et al., 2007). Conversely, after the dive, when the divers out of the water, they had resumed a sitting posture, the water hydrostatic pressure had disappeared, and their plasma volume can be assumed to be lower than at baseline period (Castagna et al., 2013). All these changes support a decreased vagal outflow, with a lowered HF spectral power, and an increased LF/HF. Thus, the indirect findings of autonomic changes with OC dives in this study appear fully consistent with the results obtained in head-out immersion studies (Mourot et al., 2007) and confirm the earlier results of Chouchou et al. (2009) which were measured in a less homogenous sample of subjects.

Besides temporal and harmonic spectral analysis of HRV, we performed short-term fractal analysis. Changes in fractal HRV organization provide information about specific autonomic settings which may be associated with potential cardiovascular disorders (Goseki et al., 1994; Krstacic et al., 2007). The FrD of a waveform represents a powerful tool for transient detection. The higher the FrD, the more irregular the signal is. Since the release of the first algorithm several papers have demonstrated its usefulness and validity in heart rate studies (Henriques et al., 2020). There is emerging consensus that a FrD value higher than 1.5 may indicate an imbalance between the sympathetic and parasympathetic arms of the ANS (Struzik et al., 2004). A higher than usual value may favor a syncope hazard, especially if FrD is greater than 1.8 and the HF index is low (Butler et al., 1993). ApEn quantifies the regularity of time series, in other words it characterizes randomness (Henriques, et al., 2020). The more regular and predictable the R-R interval series, the lower the value of ApEn. The significant decrease of the *α*1 fractal exponent during the descent was found to reflect a co-activation of both sympathetic and vagal autonomic arms (Tulppo et al., 2005), and in OC diving, neither FrD or the approximate entropy (ApEn) was significantly modified.

In the second part of our study, we analyzed the same classic HRV parameters in CCR diving. In a dive with similar (although not identical) nitrogen exposure (dose), body position, physical effort, respiratory effort, and body temperature conditions, the HRV changes were either not observed, blunted, or delayed. However, using fractal analysis, we observed a significant increase in FrD and a non-significant increase in ApEn compared to OC diving.

Although far from the threshold for pathological event, the observed increase in FrD while diving with a CCR reflects a shift toward a more random heart rate control. Likewise, the increase in approximate entropy (ApEn) stayed within accepted physiologic limits (Huikuri et al., 2003; Krstacic et al., 2007; Voss et al., 2009). Together the different analysis consistently indicates that during descent, at depth, and during the ascent toward surface the cardiovascular system was operating at a lower level of variability which might increase the hazard burden of known or unknown cardiac disease (Butler et al., 1993).

It thus seems that CCR diving provokes a different HRV response in divers than OC diving.

While the diving exposure obviously is different in many aspects, factors which would be suspected to influence HRV (immersion, physical effort, and body position) by direct underwater supervision by at least one of the authors. Two major differences can be defined though: The CCR dives were twice as long as OC dives and implied breathing of a constant PpO2 which was more than 1.5 times higher than the PpO2 during the bottom phase of the OC (1.3 ATA vs. 0.8 ATA). Because the HRV differences were already observed during the first phases of the dives (descent, 15 min bottom time; see Figure 2), we postulate that the dive time is not a determining factor in this different HRV response, and that a putative role of oxygen must be considered, as suggested by Chouchou et al. (2020).

This is of importance since chemoreceptor activity is one of the many factors capable of influencing short-term HRV (Lundell et al., 2021).

Although the trends of the different parameters studied are identical, the indices of higher parasympathetic modulation of cardiac activity appear more pronounced in CCR than OC dives. Indeed, the increased partial pressure in oxygen is known to decrease heart rate and increase the HF power of HRV, suggesting a chemoreflex-induced increase in vagal activity (Lund et al., 2003). After the end of hyperoxic exposure, the shift of power spectral distribution of HRV toward a pattern of increased cardiac sympathetic activity was found after 30 min out of water and reflecting a resuming of baseline autonomic balance (Gole et al., 2011). The same pattern has been observed in athletes and correlated with activity of catalase, superoxide dismutase (SOD), thiobarbituric acid reactive species (TBARS), oxidative modification products, middle mass molecules, and ligand forms of hemoglobin (Ielisieieva et al., 2007). Improvement of sympathovagal balance has also been proposed among OC nitrox divers (Zenske et al., 2020a).

Our study has several strengths and limitations. First, divers were chosen to be biometrically as closely resemblant to one another as possible, in biometry, general health status and activity level. Secondly, diving exposures were not “at free will” but controlled in depth, duration, and physical activity. Thirdly, on top of the usually studied HRV parameters, we applied fractal analysis as a measure of complexity, which has not previously been reported in this setting.

Limitations of our study can be summarized in three main points.

The number of subjects being low, there may be a concern for reliability and power of the study results. Statistical power is hard to calculate as up until now, no comparison between HRV differences in OC and CCR diving has been made.

Secondly, there are major differences between OC and CCR diving in terms of breathing mechanics, as the breathing valves of a CCR have very low resistance, as opposed to those in OC. However, increased respiratory resistance is only seen at high ventilation rates, such as associated with exercise above 150 watts (Butcher et al., 2006), which was unlikely in both dives. Indeed, exercise intensity inferred from surface equivalent air consumption corresponded to a low- to moderate-intensity energy expenditure (Buzzacott et al., 2014). More, by adapting the limits of frequency analysis, we limited the influence of respiratory sinus arrhythmia related to work of breathing in both groups.

Thirdly, although the body temperature of CCR divers was maintained during the whole experiment, the mechanism of thermoregulation is by far more complicated than just keeping the body warm. Therefore, it must be acknowledged that the difference of thermic control between OC and CCR dives [passive factors: thermoneutral pool water, limitation of convective and conductive heat loss using wetsuit protection *vs*. both passive protection and active heat generation (battery-powered heating vest)] may have played a significant role. Indeed, it has been shown that body cooling and water immersion lead to autonomic co-activation and cumulated strengthening of parasympathetic cardiac control (Mourot et al., 2008). However, to further ascertain that the thermal status was not among the most influencing factors, a specific analysis of the very low-frequency power (VLF) component of the HRV, which is known to reflect thermoregulation to ambient temperature changes (Thayer et al., 1997) has been performed. Since we observe no significant difference from baseline after the dive in both groups (*p* = 0.12), thermal status was, in our view, not a major factor. However, it would have been better had we been able to conduct the CCR dives in the same indoor pool setting.

## Conclusion

These results add further evidence for marked changes in autonomic cardiac activity during and after scuba diving. An increase of parasympathetic influence is observed during descent and bottom stay. This balance is reversed after emersion and resuming of normobaric breathing. Cardiovascular baroreflex stimulation would support a large part of these changes. However, it seems that part of these changes might depend on chemoreflex deactivation/activation when breathing higher oxygen pressures than achieved by breathing normobaric air. Further research on that topic is encouraged.

## Data Availability Statement

The raw data supporting the conclusions of this article will be made available by the authors, without undue reservation.

## Ethics Statement

The studies involving human participants were reviewed and approved by the Haute Ecole Bruxelles-Brabant, Brussels, Belgium (B200-2020-088). The patients/participants provided their written informed consent to participate in this study.

## Author Contributions

All the authors contributed to at least three of the four major components of a study. The author hereby declares that the present manuscript is the result of original work by the authors, all co-authors have given their permission for publishing the manuscript, have read the submission, and agree to be listed as co-author the manuscript is currently not under submission review with another scientific journal.

## Conflict of Interest

The authors declare that the research was conducted in the absence of any commercial or financial relationships that could be construed as a potential conflict of interest.

## Publisher’s Note

All claims expressed in this article are solely those of the authors and do not necessarily represent those of their affiliated organizations, or those of the publisher, the editors and the reviewers. Any product that may be evaluated in this article, or claim that may be made by its manufacturer, is not guaranteed or endorsed by the publisher.
